# Osteoimmunomodulation role of exosomes derived from immune cells on osseointegration

**DOI:** 10.3389/fbioe.2022.989537

**Published:** 2022-08-19

**Authors:** Yunchao Xiao, Yanshu Ding, Jingwen Zhuang, Ruoyue Sun, Hui Sun, Long Bai

**Affiliations:** ^1^ College of Materials and Textile Engineering, Jiaxing University, Jiaxing, China; ^2^ Nanotechnology Research Institute, Jiaxing University, Jiaxing, China; ^3^ Engineering Research Center for Biomedical Materials of Ministry of Education, College of Materials Science and Engineering, East China University of Science and Technology, Shanghai, China; ^4^ Department of Orthopaedic Surgery, Shanghai Jiao Tong University Affiliated Sixth People’s Hospital, Shanghai, China; ^5^ Institute of Translational Medicine, Shanghai University, Shanghai, China

**Keywords:** osseointegration, osteoimmunomodulation, immune cells, exosomes, macrophages

## Abstract

Despite the high success rate of biomedical implants adopted clinically, implant failures caused by aseptic loosening still raise the risk of secondary surgery and a substantial economic burden to patients. Improving the stable combination between the implant and the host bone tissue, achieving fast and high-quality osseointegration can effectively reduce the probability of aseptic loosening. Accumulating studies have shown that the osteoimmunomodulation mediated by immune cells mainly dominated by macrophages plays a pivotal role in osseointegration by releasing active factors to improve the inflammatory microenvironment. However, the mechanism by which osteoimmunomodulation mediates osseointegration remains unclear. Recent studies have revealed that exosomes released by macrophages play a central role in mediating osteoimmunomodulation. The exosomes can be internalized by various cells participating in *de novo* bone formation, such as endothelial cells and osteoblasts, to intervene in the osseointegration robustly. Therefore, macrophage-derived exosomes with multifunctionality are expected to significantly improve the osseointegration microenvironment, which is promising in reducing the occurrence of aseptic loosening. Based on this, this review summarizes recent studies on the effects of exosomes derived from the immune cells on osseointegration, aiming to provide a theoretical foundation for improving the clinical success rate of biomedical implants and achieving high-quality and high-efficiency osseointegration.

## 1 Introduction

Biomedical implants for dentistry and orthopedics have been widely used, with more than 1.5 million patients receiving implant replacements annually (Alghamdi et al., 2014). With the development of social productivity and the improvement of medical and health conditions, the average lifespan expectancy has increased significantly, and the global aging trend has become much more apparent. Accordingly, the medical demand for implant replacement will show an accelerated upward trend. However, an 8-years investigation showed a 2–3% failure rate for dental implants (Pjetursson and Heimisdottir, 2018). Implant failure leads to the implementation of a second operation, which prolongs the patient’s recovery period and brings patients huge mental pain and inconvenience. Despite the infection around the implant, over half of implant failures are due to aseptic loosening (Barnsley and Barnsley, 2019). Therefore, the ideal implant should avoid aseptic loosening, which can effectively promote osseointegration at the interface after implantation and prolong the service life of the implant.

Osseointegration was initially defined as “the direct structural and functional connection between the bone tissue and the implant surface” (Guglielmotti et al., 2019). In 2012, Zarb et al. redefined osseointegration as “a time-varying healing process that progressively achieves rigid binding of material to bone tissue and stable retention in bone during functional loading at the interface”. This definition explains in more detail that osseointegration is a complex process that changes dynamically over time, mainly through four overlapping and synergistic stages: early clot formation, immune response, angiogenesis, and osteogenesis (Bai et al., 2021a). The immune cell-mediated inflammatory response begins immediately, usually within 12 h, and completes within 7 days after implantation. Immune cells include neutrophils, mast cells, and macrophages. Among them, macrophages play a crucial role in the immune response and are the primary effector cells of the inflammatory response (Bai et al., 2021b). Advances in osseointegration research have pinpointed the pivotal role of immune cells, especially macrophages, in manipulating angiogenesis and osteogenesis to achieve a fulfilled bone formation upon the implant surfaces (Bai et al., 2018). However, the underlying mechanism of the cross-talk between immune cells and bone formation-related cells is still unclear.

Exosomes are multivesicular bodies formed by intracellular lysosomal particles secreted by various cells such as bone marrow stem cells and macrophages (Jeppesen et al., 2019). Accumulated studies have depicted that exosomes play pivotal roles in tissue regeneration and multiple disease treatments (De Toro et al., 2015). Recent studies showed that exosomes secreted by immune cells could dominate bone formation through osteoimmunomodulation (Wei et al., 2019). Exosomes exist as a cell-free therapy and have superior advantages that other strategies do not possess, such as smaller size (40–160 nm), long circulatory half-life, low immunogenicity, accessible to cross the blood-brain barrier, easy production and storage, and no tumorigenicity, conferring it as an auspicious choice for regenerative medicine and biomedical treatment (Butler, 2002). However, at the same time, due to the heterogeneity of vesicles and the lack of specific targeting mechanisms, exosomes are often trapped in non-specific tissues, resulting in the lack of targeting ability in the process of regulating osseointegration of exosomes (Lai et al., 2014, Zhang et al., 2021). This review aims to summarize the role of exosomes secreted by multiple immune cells, especially the macrophages, in manipulating *de novo* bone formation and osseointegration; Meanwhile, we overview the effect of surface modification of biomaterials on the functionality of exosomes derived from immune cells.

## 2 Exosome

### 2.1 Source and description of exosomes

Exosomes are lipid-bound vesicles secreted by cells into the extracellular fluid. They are also called intraluminal vesicles (ILVS). They are mainly derived from vesicles formed by intracellular lysosomal granules. All cells can secrete exosomes regardless of normal or pathological conditions. They naturally exist in body fluids, including plasma, urine, and semen, which usually have a diameter between 30–150 nm. Specifically, the early cell plasma membrane germinates inward, causing endoderm proteins and other components to be phagocytosed, and in the process, it grows into a polycystic body (MVB). MVB is involved in the endocytosis and transportation of cellular material. Finally, part of the MVB is sent back to the lysosome, and part of the plasma membrane is fused and released into the extracellular fluid, of which exosomes are a part (Doyle and Wang, 2019). Ultracentrifugation is a traditional, efficient and reproducible method for purifying and extracting exosomes, which is suitable for purifying exosomes from cell culture media (Caradec et al., 2014). In their study, Webber et al. found that the use of ultracentrifugation on a sucrose pad was the best method to obtain high-purity exosome fractions suitable for purification of exosomes from cell culture media from urine and serum. The extracted exosomes are of low purity. While some commercial kits are used to isolate and study exosomes for various purposes, the kits are less time-consuming, less technically sensitive and more compatible with a limited number of biological samples compared to UC, and do not Special equipment is required (Helwa et al., 2017). One of the ways to label exosomes is by identifying the presence of proteins secreted by the exosomes, the most common and easiest of which is by immunoblotting, by biomarker exosome fractions compared to total cell lysates However, due to the huge differences in plasma protein concentrations and extremely low levels of other proteins such as cytokines, biomarkers are only used as prognostic indicators. Mass spectrometry is an important tool for assessing the quality of exosomes because of its repeatable and accurate quantification, strong ability to identify signals, and low signal-to-noise ratio (Rifai et al., 2006; Xu et al., 2020). The optimal storage temperature for long-term storage of exosomes used in clinical and research is -70°C. For short-term storage, exosome proteins will be degraded above 37°C (Lee et al., 2016).

### 2.2 Composition and function of exosomes

The contents of exosomes include receptors, transcription factors, enzymes, extracellular matrix proteins, lipids, nucleic acids, and various protein complexes on the surface. The analysis of exosome protein composition indicated that some proteins are common in all exosomes, and some belong to non-specific protein types. Furthermore, the lipid content of exosomes is cell-specific or conservative (Mashouri et al., 2019). The specific behavior of lipids in MVB is mainly determined by the nature of the hydrophobic tail (Stoorvogel et al., 2002). A common feature of different types of exosomes is the expression of their adhesion molecules. Adhesion molecules and adhesion receptor systems participate in various critical cellular processes such as cell growth and differentiation. Types of adhesion receptors on the cell surface are divided into the integrin family, immunoglobulin super protein family, cadherin family, and selectin family (Frenette and Wagner, 1996). Exosomes also contain a unique protein composition, including proteins such as Tsg101 (Guo et al., 2015). Tsg101 is a component of the endosome sorting complex (ESCRT) necessary for the transport mechanism, which plays a direct role in releasing nucleocapsids from the polycystic endosome into the cytoplasm (Luyet et al., 2008). Exosomes are also rich in lipids related to lipid rafts, such as sphingolipids and cholesterol (Wubbolts et al., 2003). Exosomes also contain functional biological enzymes.

**FIGURE 1:**
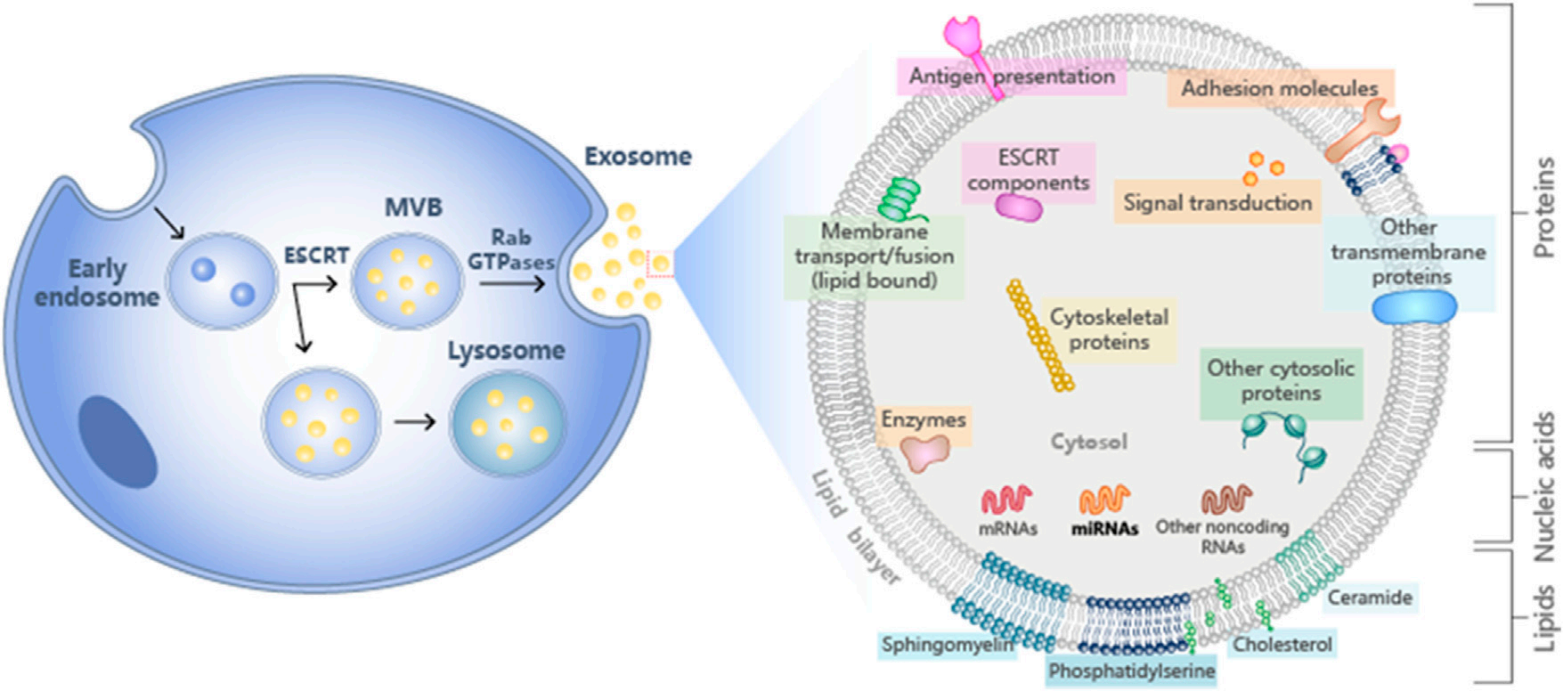
Schematic diagram of the composition of exosomes.

The function of exosomes is to eliminate unfavorable biological molecules and play a role in immune surveillance. Exosomes containing microbial components can promote antigen presentation and macrophage activation (Schorey and Bhatnagar, 2008). Recent research has focused on the functionality of exosomes in antigen presentation. Exosomes promote T cell immunity during bacterial infection and are an essential source of extracellular antigens (Smith et al., 2017). Exosomes also play an important role in immune and metabolic regulation (Figure 1).

## 3 Osseointegration

### 3.1 The importance of osseointegration

**FIGURE 2:**
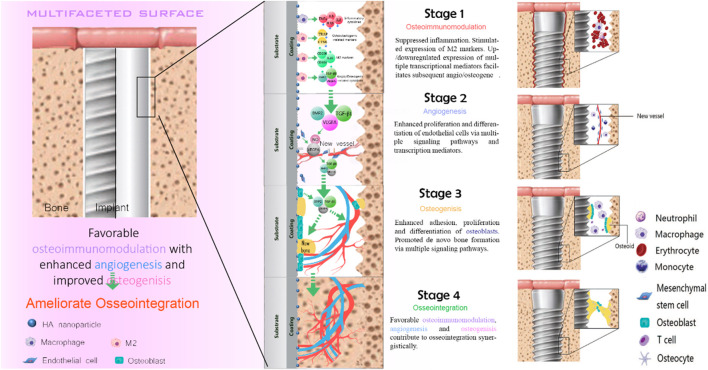
Schematic diagram of osseointegration.

With the continuous development of biomaterials, the emergence of biomedical implants provides an alternative solution for patients with bone injury. The implantation of the implants is divided into two stages. The first stage is the placement of artificial implants into the injured site within the bone tissue. After 3–6 months of bone regeneration, the implant and the alveolar bone complete tight osseointegration. This process requires sufficient bone mass to maintain strong support mechanically. Despite the favorable clinical success rate, the main reasons for implant failure are postoperative infection and aseptic loosening caused by poor osseointegration. Osseointegration is a sophisticated process that involves blood clots formation, the immune response of immune cells, angiogenesis of endothelial cells, and new bone formation of the bone-related cells (Figure 2).

### 3.2 Early blood clot formation

The implantation of the implant will cause a certain degree of surgical damage to the blood vessels around the implant. This process usually lasts from a few minutes to a few hours. Blood will directly contact the implant, and then plasma proteins will be adsorbed on the implant’s surface. Platelets promote hemostasis through activation, adhesion, and aggregation (Periayah et al., 2014). Platelets are activated at the interface, transforming the main platelet integrin ανβ3 from resting to the active conformation. The activated integrin continues interacting with the platelets (Terheyden et al., 2012). Phase interaction can lead to further activation of platelets. Activated platelets can mediate the expression of GPIIb/IIIa (or integrin αIIbβ3), glycoprotein-1 (TSP-1), and P-selectin (Agarwal and García, 2015). αIIbβ3 mediates platelet aggregation, forming thrombus, blocking blood vessel leakage, and at the same time binding to fibrin to generate more outward signals, leading to further platelet activation (Terheyden et al., 2012; Agarwal and García, 2015). TSP-1, thrombospondin-1, contributes to the activation of TGF-β1, a transforming growth factor. TGF-β1 plays an essential role in angiogenesis and tissue fibrosis (Ahamed et al., 2009). TSP-1 can induce platelet aggregation (Losic et al., 2015). The interaction between p-selectin and the ligand stabilizes the initial interaction of GP IIb/IIIa-fibrinogen, thereby forming numerous stable platelet aggregates (Berger et al., 1998). The expression of the above three signal molecules will cause local thrombin to increase (Guihard et al., 2015). Thrombin can convert fibrinogen into insoluble fibrin clots (Colnot et al., 2007).

### 3.3 Immune response

After the blood clot forms, the immune response starts immediately. The immunization process begins about 12 h after surgery and lasts 7 days. This process is mediated by immune cells residing in the bone marrow, including dendritic cells, T cells, B cells, macrophages, neutrophils, and mast cells. In the early stage, the inflammatory response is mediated by neutrophils. Neutrophils kill bacteria through reactive free radicals (oxygen-free radicals and hydroxyl radicals, chlorine-free radicals, and hypochlorite), and because this method is non-specific, It may also lead to the loss of surrounding healthy tissue. At the same time, it also secretes digestive enzymes, further aggravating the damage to adjacent tissues. Early period inflammation plays a decisive role in the future development of the immune defenses (Marchi et al., 2013). Neutrophils are in the terminal differentiation stage and have a short life span and will eventually be replaced by macrophages and lymphocytes. There is a special kind of macrophages in bone tissue called bone macrophages. Cytokines secreted by macrophages, such as BMP-2, TNF-α, and OSM, can regulate bone formation (Ferrarelli, 2013). At the same time, macrophages have multiple types, among which macrophages that are not activated are called the M0 type. After activation and differentiation, M1 and M2 are two phenotypes (Guastaldi et al., 2013). IL-6, IL-1β, and TNF-α expressed by M1 macrophages can promote inflammation, while IL-10 and CD206 expressed by M2 macrophages can inhibit inflammation (Chernousova and Epple, 2013). In addition, M2 macrophages can also manipulate a favorable immune microenvironment through cell growth factors such as BMP-2 and TNF-α (Marchi et al., 2013).

### 3.4 Angiogenesis

Angiogenesis at the bone-implant interface is considered a prerequisite for osseointegration. The process is also initiated within 24 h, accompanied by the immune response, which refers to the production process of new capillaries, including two forms of blood vessel formation and angiogenesis. Angiogenesis is the migration and aggregation of angioblasts (including progenitor cells of endothelial cells and blood cells) to a specific site to form the first batch of primitive blood vessels. Then new blood vessels are generated through a series of processes such as EC sprouting, migration, multiplication, vascular anastomosis, and pruning (Sivaraj and Adams, 2016). Angiogenesis is a key process that supports new bone formation in osseointegration. It also plays a crucial role in fracture repair and healing. Changes in local blood vessels are also closely related to many bone diseases such as osteoporosis (Brammer et al., 2012). Studies have shown that hypoxia and some growth factors are the main signals that drive angiogenesis in bone. These signals not only affect the angiogenesis process but also affect the subsequent osteogenesis process. The primary regulator of VEGFA in angiogenesis is produced and secreted by hypertrophic chondrocytes (Brammer et al., 2012). VEGFA can induce endothelial cell migration and proliferation by inducing its receptor VEGFR2. In addition, VEGFA isoforms can activate the β-catenin signaling pathway to promote angiogenesis and bone formation in bone tissue.

### 3.5 New bone formation

The formation of new bone is accompanied by angiogenesis. Osteoblasts and osteoclasts are the primary effector cells in the process of osteogenesis. There will be bone morphogenetic protein (BMP) in the wound at first. After the implant has initial stability, it will rub against the primary bone and be fixed in the damaged site. The amount of contact and the strength are closely related to the bone density of the host bone tissue. One week after implantation, the new bone is connected with the primary bone to form a secondary bone contact, and the first piece of braided bone is produced (Terheyden et al., 2012). The osteogenic process is closely related to the communication and cooperation between osteoblasts and osteoclasts. Osteoclastic bone resorption allows TGF-β and BMP to be released from the cell matrix, activating more osteoblasts to secrete the growth factors (Matsuo and Irie, 2008). Osteoblasts secrete the collagen matrix, which then rapidly mineralizes. Subsequently, the osteoclasts remove the braided bone and replace it with the layered bone. Consequently, the implant’s surface is covered by mature layers of bone, and the osseointegration is completed immediately (Hoppe et al., 2011).

### 3.6 The importance of immune response in osseointegration

Since the implant is a foreign body, implantation will inevitably bring about an inflammation-driven process on the implant’s surface and its vicinity. This process is manipulated by immune cells, and the early immune microenvironment determines the result of osseointegration (Bai et al., 2018). The immune response can affect bone formation by modulating the functionality of osteoblasts. That is, the immune system and the skeletal system are inextricably linked. The immune system can regulate bone tissue repair and regeneration through osteoimmunomodulation.

## 4 The role of immune cells derived exosomes in osseointegration

Wei et al. discovered that titanium nanotube implants loaded macrophage-derived exosomes greatly improved the production of ALP and BMP-2 protein of earl osteoblast. The combination of exosomes released from macrophages can enhance bone formation. Exogenous miR-5106 from M2 macrophages can stimulate bone marrow stem cells to differentiate into osteoblasts. Exosomes enhanced the proliferation of MC3T3-E1 cells compared to the control group in a study evaluating the influence of exosomes on the osteogenic differentiation of MC3T3-E1 cells. Exosomes also induce the expression of cellular type I collagen (present in bones, accounting for more than 90% of bone organic matter) and Runx2, which further facilitate bone formation (Komori, 2010). Moreover, exosomes can increase the adhesion and proliferation of bone marrow mesenchymal stem cells, and immobilized exosomes can increase the expression of the cell-derived factor (SDF-1) gene (Wang et al., 2020). Briefly, exosomes play a vital function in the body’s immune system (Zhang et al., 2021).

### 4.1 Neutrophils and mast cell

Neutrophils are the most abundant white blood cells in the blood circulation and are also one of the first immune cells recruited around the implant. Neutrophils are derived from pluripotent stem cells in the bone marrow. After differentiation and development in the bone marrow, neutrophils’ immunity is immediately innate. If the implant’s surface is infected, IL-8 cytokines related to inflammation will respond and play an essential role in the activation of neutrophils. After being recruited around the implant, the first role played by neutrophils is phagocytosis, which can swallow part of bone debris and microorganisms. Subsequently, phagocytosis triggers the production of effective biocides such as reactive oxygen species such as hydrogen peroxide and superoxide free radicals. Neutrophils are the first and most important line for defending against microbial invasion. Mast cells are also derived from pluripotent bone marrow-derived stem cells. Mast cells can express various surface receptors, prompting them to cooperate with other immune cells to complete the immune process. At the same time, mast cells can produce numerous pro-inflammatory cytokines to affect cell recruitment and promote immunity (Mekori, 2004).

Neutrophil-derived exosomes are composed of a variety of proteins and RNAs. Under the stimulation of neutrophil exosomes, the acellular glycoprotein TSP-1 protein, which is related to blood clot formation and angiogenesis, is strongly expressed. The TSP-1 protein mainly exists in the platelet alpha granules, and the effect of TSP-1 on platelets is still unclear. However, studies have shown that TSP-1 plays a role in the platelet-endothelial interaction. The cAMP regulatory pathway has been learned to regulate platelet function at the injury site. Studies have shown that TSP-1 does not directly affect the blood clot formation process by activating platelets directly, but indirectly affects coagulation by reducing platelet inhibitors. TSP-1 triggers CD36-dependent signals to reduce the sensitivity of platelets to PGE-1 stimulated by endothelial-derived mediators, thereby weakening their ability to inhibit platelets (Roberts et al., 2010).

The contribution of mast cell exosomes is manifested in the transfer of molecules between cells, which promotes the communication between molecules. The exosomes secreted by mast cells not only affect the process of osseointegration through the components of the exosomes themselves but also have close connections with a variety of cell types. Mast cell-derived exosomes can activate B and T lymphocytes (Skokos et al., 2001). Mast cells are located close to blood vessels and assemble into piles, contributing to the secretion of clotting factors during inflammation, thus playing an immunological role. After activation, mast cells-derived exosomes induce PA1-1 expression in endothelial cells by the prothrombinase complex (Al-Nedawi et al., 2005).

### 4.2 Macrophage

Macrophages are immune cells differentiated from hematopoietic stem cells. The cells that reside in blood vessels are called monocytes. Macrophages immigrate outside blood vessels and participate in innate immunity in vertebrates through phagocytosis to eliminate pathogens. Compared with Neutrophils, which live only a few months, the lifespan of macrophages can range from several months to several years. Macrophages not only have a phagocytic effect but are also responsible for the clearance of neutrophils after apoptosis. Macrophages that exist in bone tissue are called bone macrophages.

The exosomes derived from macrophages contain many tiny mRNA and micro RNA. mRNA is a type of endogenous, small molecule, and non-coding RNA that can bind to target genes, control the expression of target genes in reverse, regulate the production of cytokines, and affect cell functions. At the same time, mRNA itself also has specific functions as listed (Table 1)

**TABLE 1:** Composition and function of macrophage exosomes.

Type	Name	Function
mRNA	miR-222	Osteogenesis,anti-angiogenesis
mRNA	miR-223	Immune process
mRNA	miR-155	Endothelial cell function
mRNA	mmu-circ-000359	M1-M2

mRNA is critical in the differentiation of macrophages into the M2 type. LPS may trigger the production of M1 macrophage markers, but when mmu-circ-0001359 exosomes were added, the expression of M1 macrophages was considerably decreased, whereas M2 macrophages were significantly elevated. As a result, when activated by LPS, mmu-circ-0001359 can enhance the transition of M1 macrophages to M2 macrophages. Through luciferase labeling and analysis to explore the mechanism of microRNA affecting the phenotype of macrophages, it is found that both FoxO1 and miR-183–5P are the targets of mmu-circ-0001359 (Shang et al., 2020). FoxO1 can be used as a regulator to affect the phenotype of macrophages. miR-5p controls the NF-κB signaling pathway in M1 macrophages via the targeted gene PPP2CA and stimulates the production of pro-inflammatory cytokines IL-1, IL-6, and TNF-α (Guo et al., 2020).

Exosomes derived from macrophages can promote macrophages’ activation and regulate cytokine production *in vitro*. Due to the increased demand for cell metabolism during the inflammatory reaction and the reduction of cell metabolic substrates, it is easy to cause tissue cell hypoxia (Eltzschig and Carmeliet, 2011). Under hypoxia/serum deprivation h/SD conditions, BMSC activity decreases, and apoptosis of BMSCs increases. Meanwhile, under h/SD conditions, exosomes derived from M1 macrophages can be released to bone marrow mesenchymal stem cells. miR-222 in the exosomes induces bone marrow mesenchymal stem cell apoptosis, thereby regulating bone formation (Qi et al., 2021). miR-222 also plays an important role in angiogenesis and is the most expressed gene in endothelial cells (Suárez et al., 2007). miR-222 is highly expressed in EC and VSMC. miR-222 inhibits endothelial cell migration, proliferation, and angiogenesis *in vitro* by targeting the stem cell factor receptor c-Kit and indirectly controlling the production of nitric oxide synthase. Two enzymes, Dicer and Drosha, mediate two processing routes that generate mRNA. miR-222 can act on c-Kit and e-NOS, two important pro-angiogenic regulators. Overexpression of miR-222 can indirectly reduce the expression of nitric oxide synthase. This affects endothelial cell functions, including inhibition of angiogenesis and wound healing. However, miR-222 can also promote blood vessel growth by reducing the expression of c-Kit in hematopoietic progenitor cells (Kuehbacher et al., 2008).

miR-223 is also enriched in exosomes produced by macrophages under hypoxic conditions (Krzywinska and Stockmann, 2018). The hypoxia response is regulated by the hypoxia-inducible factor HIF and the HIF signaling pathway, which plays an important role in the recruitment of immune cells (Zhu et al., 2019). miR-223 induces the production of more exosomes and locally activates the immune systems. In the inflammatory phase, the production and release of exosomes are abundant. As an innate immune regulatory factor, miR-223 can promote the differentiation of granulocytes and inhibit the differentiation of macrophages. The expression of miR-223 is highest in the bone marrow cavity, and it plays an important role in the differentiation of granulocytes and macrophages in the differentiation of bone marrow. miR-223 is a negative regulator of neutrophil activation and plays an important role in the regulation of neutrophil function. Overexpression of miR-223 will reduce the activity of the pyrin domain of the NLR family by directly targeting NLRP3, thereby inhibiting NLRP3-dependent production of IL-1β. Neutrophils are the key mediator of MTD-induced ALI, and miR-223 can inhibit the inflammatory activity of NLRP3 by inhibiting the number of Ly6G^+^ neutrophils. Increased levels of miR-223 inhibit the expression of APAP-induced liver injury target gene IKK-ɑ and inhibit the TLR-NF-κB inflammatory pathway. In addition, miR-223 can promote the polarization of macrophages to the anti-inflammatory M2 type by directly targeting Pknox1. LPS can reduce the expression of miR-223 through TLR4 and TLR3 activation. Meanwhile, an increase in the protein level of the STAT3 gene in the target gene of miR 223 leads to a selective increase in the IL-6 gene and the IL-1β gene, further increasing the inflammatory reaction. The IL-6 canonical signal pathway regulates the decline of miR-223, which leads to the increase of Ras homologous gene family and Rho-B expression, induces the activation of NF-κB and MAPK signal pathways, and promotes TNF-α, IL-6, and IL-1β stimulated by LPS. In addition, miR-223 can also cause changes in the expression level of NLRP3 and inhibit the response of IL-1β to LPS stimulation (Yuan et al., 2018).

Macrophage exosomes can guide macrophage activation and transformation by driving the reprogramming of macrophages. Exosomes deprived of M2 macrophages can guide M1 macrophages to reprogram into M2 macrophages. INEOS is a typical M1 marker, while Arg-1 is a typical M2 marker. After treatment with exosomes derived from M2 macrophage, the expression of NOS (nitric oxide synthase) and arginine were significantly reduced and increased, respectively, while the results were opposite after treatment with exosomes derived from M1 macrophage. The western blot method also proved the change in the expression pattern of exosomes deprived of macrophage-specific markers. The experiment also proved that after treatment with exosomes derived from M2 macrophage, the local switching of exosomes deprived of M1 macrophage successfully promoted the re-epithelialization of exosomes deprived of M2 macrophage (Kim et al., 2019). Both forms of exosomes reduced the expression of the inflammatory cytokines such as IL-1β and TNF-α when compared to natural bone marrow macrophages treated with control PBS.

Exosomes derived from macrophages can create a favorable immune microenvironment. The major histocompatibility complex molecule and the costimulatory molecule CD86 are present on the surface of exosomes, which may enhance the immune response. Exosomes deprived of macrophages also contain a transmembrane protein lysosomal-associated membrane protein 2 (LAMP 2) and a cytosolic protein B actin, which is often detected in exosomes (Bouchareychas et al., 2020). Previous studies have shown that exosomes infected with inflammation may induce antigen-specific T cell responses. Exosomes from infected cells induce the expression of IFN-γ in CD8^*+*^ and CD4^+^ T cells, and CD69 expression is enhanced, reflecting the ability of exosomes to act as antigens to stimulate these two T cells populations.

Since macrophages can promote the angiogenesis process of endothelial cells, the entire macrophage population is defined as the pro-angiogenic cell population (Novoselova et al., 2015). After the inflammatory period begins, the immune microenvironment changes, which stimulates the polarization of macrophages (Zhang et al., 2017). Due to ischemia sexual and hypoxic stress, a large number of chemokines and pro-inflammatory cytokines are produced (including interferon IFN-γ, bacterial lipopolysaccharide (LPS), interleukin (IL)-1β, and tumor necrosis factor-α (TNF-α)), induces polarization of M1 macrophages. miR-155 is a regulator of M1 macrophage differentiation and one of the miRNAs with high content of M1 macrophages (Ortega et al., 2015). M1 type macrophage exosomes can transfer miR-155 into endothelial cells, and miR-155 can promote plasma soluble intercellular adhesion molecule-1 (SICAM -1) and soluble vascular cells by inhibiting the Akt-Enos pathway signaling pathway. miR-155 also targets the RAC1-PAK2 signaling pathway. Both RAC1 and PAK2 are proteins involved in angiogenesis. They control cell migration and cell-cell connection in endothelial cells, thereby regulating vascular permeability.

Macrophages and endothelial cells can establish contact through the cross-talk of exosomes. The communication process between macrophages and endothelial cells is crucial to the immune process and angiogenesis. Macrophage exosomes control the migration process of endothelial cells and then control the transport of integrins. The transport process of integrins includes internalization, circulation, and lysosomal degradation and mainly acts on connecting cells and neighboring cells or cells and their extracellular matrix. Macrophage exosomes induce integrin β1 lysosomal degradation and internalization and meanwhile promote integrin β1 ubiquitination and inhibiting the activation of the MEK/ERK pathway. The signaling pathway responds to extracellular stimuli and converts the signal into a cellular process by extracellular binding ligands to specific transmembrane receptors. The MEK/ERK signaling pathway is mainly responsible for regulating the phosphorylation sites of activity. Integrin β1 accumulates in the perinuclear area and no longer returns to the plasma membrane (Raman and Cobb, 2003; Shaul and Seger, 2007; Lee et al., 2014; Li et al., 2022). At the same time, integrin β3 is not affected by the action of the exosome.

**FIGURE 3:**
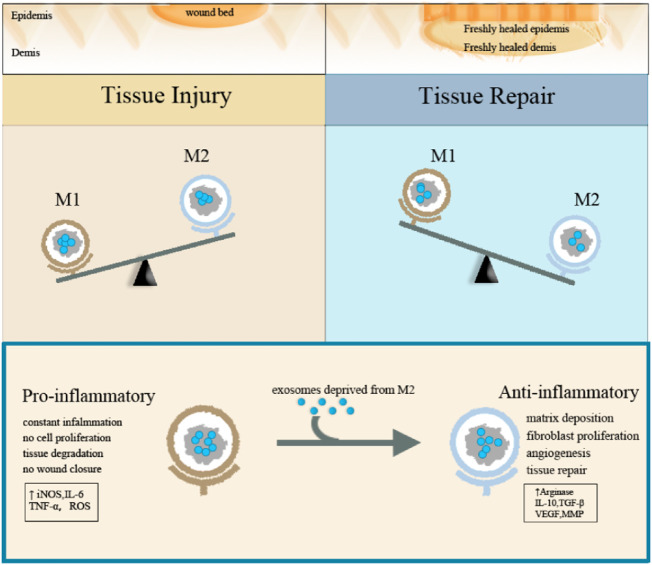
Schematic diagram of the function of macrophage exosomes.

M1 type macrophage exosomes can transmit pro-inflammatory signals and establish a local immune microenvironment. M1-type macrophages can enter the lymph nodes and are taken up by macrophages and dendritic cells. Exosomes deprived of M1 macrophages will induce macrophages to produce high levels of pro-inflammatory cytokines such as IL-6, IL-12, IFN-γ, TNF-α, and proteases, while exosomes deprived of M2 macrophages will induce macrophages to produce M2 phenotypes with low IL-6, low TNF-α, low IL-12, and high IL-4 and IL-10 (Figure 3).

Exosomes derived from macrophages significantly impact the proliferation and differentiation of mesenchymal stem cells. The exosomes produced by M1 macrophages promote the proliferation, osteogenic and adipogenic differentiation of bone marrow mesenchymal stem cells, and the exosomes produced by M2 macrophages can significantly boost the osteogenic differentiation of mesenchymal stem cells. miR-5106 is abundantly expressed in M2 macrophage exosomes. miR-5106 induced more obvious alkaline phosphatase activity. Moreover, exosomes derived from M2 macrophages have increased bone formation-related genes (ALP, osteopontin, RunX2, BMP-2, and BMP-7) (Chen et al., 2020). The three types of macrophage exosomes, M0, M1, and M2, all have inhibitory effects on cartilage differentiation (Bouchareychas et al., 2020; Xia et al., 2020).

### 4.3 T cell and B cell

Lymphocytes are divided into T lymphocytes, B lymphocytes, and natural killer cells (NK cells). They are the main cell group that constitutes the body’s immune system and participate in body immunity and humoral immunity. T lymphocytes are made in the bone marrow and mature in the thymus, whereas B lymphocytes are synthesized in the bone marrow and mature in the thymus. T cells and B lymphocytes proliferate in the thymus and are then distributed to the immunological organs and tissues throughout the body via lymphatic and blood circulation to perform an immune function. (Skapenko et al., 2005; Smith-Garvin et al., 2009). Exosomes derived from T cells can regulate the internalization of T cells and endothelial cells in a CD47-dependent manner (Kaur et al., 2014).

### 4.4 NK-DC cell

Both NK cells and dendritic cells (DC) are two important components of innate immunity. NK cells are derived from bone marrow and have strong killing and immunity. Dendritic cells are formed as a result of the division of hematopoietic stem cells. NK cells mainly produce immune function through cytotoxicity and the secretion of cytokines. The cytotoxic activity is enhanced, and they express killer immunoglobulin-like receptors (CD16 and KIRS) and perforin. Factors are secreted in large quantities, expressing low levels of perforin and CD16 (Ferlazzo and Morandi, 2014). Dendritic cells are produced in large quantities, and dendritic cells are antigen-presenting cells that activate adaptive immune lymphocytes by identifying pathogens and sending pathogen information to the acquired immune system (Cooper et al., 2004). NK-DC interaction will lead to the maturation, activation, and cytokine production of both (Thomas and Yang, 2016).

Unlike other immune cells, exosomes produced by NK cells are in both resting and active states. These exosomes can express typical NK markers (CD56) when NK cells are activated and immune killer proteins (Fas-L and perforin) when NK cells are at rest status (Jiang et al., 2021). It is reported that NK cell exosomes have typical exosome proteins, such as Rab5b and MHC-I, but lack CD4 and CD8. NK cell-derived exosomes are effective for activated immune cells, which have considerable cytolytic activity and have no effect on resting immune cells (Lugini et al., 2012). Exosomes derived from NK cells under hypoxia contain higher levels of toxic proteins Fas-L, perforin, and granzyme B, and its cytotoxicity is significantly higher than that under normoxia. Meanwhile, hypoxia increases the production of exosomes (Jiang et al., 2021). DC cells exosome contain many proteins and antigens necessary to produce a robust immune response, such as MHC class Ⅰ, class Ⅱ, CD1, heat shock protein 70–90, CD9, CD63, CD81, CD11b, and CD54. Exosomes produced by DC cells activate immature CD4^+^ T cells through an indirect pathway (Giri and Schorey, 2008). CD4^+^ T plays an important role in assisting B cells to produce antibodies in immune protection, inducing macrophages to produce higher microbicidal activity, recruiting immune cells to inflammation sites and producing cytokines and chemokines, and coordinating the entire immune response (Zhu and Paul, 2008).

## 5 Outlook

As newly discovered in the past 30 years, exosomes have yet to be widely developed in terms of their functions and how they can be applied to clinical diagnosis, while the actual application process is still in its infancy. Since the average size of exosomes is less than 100 nm, the specific morphological structure cannot be displayed. It is unclear what happens when individual exosomes bind to the surface of recipient cells. However, as the practical resolving power of a microscope, lenses continue to develop and flow cytometry techniques develop, such analyses will be gradually practiced in the future. The rich composition of exosomes, special structural and morphological features, and distinct mechanisms of action are prerequisites for their role in immune responses and the promotion of intercellular communication. The role of immune cell exosomes in the four stages of osseointegration, especially the immune stage, has been confirmed by numerous studies, but whether the surface properties of different materials applied to implants affect the composition and function of immune cells exosomes has not yet been established clear, which will provide valuable guidance for improving the osseointegration.

## 6 Conclusion

Poor osseointegration at the implant interface due to the aspetic loosening will eventually result in implant failure, and a second surgery is required. Osseointegration is an integrative field in which the skeletal and immune systems interact intimately, with immune cells and their secreted exosomes functioning at all stages. The exosomes secreted by immune cells have unique immune regulation capabilities. After the hemostasis process, the immune response starts immediately, and immune cells are recruited to the area of ​​inflammation. Inflammation will promote immune cell exosomes to release the inflammatory cytokine IL-8. In addition, related to inflammation, it can regulate the phenotype of macrophages by regulating mRNAs and proteins, thereby affecting the stage of immune inflammation, mediating intercellular communication, stimulating target cells, promoting antigen presentation, transmitting pathogens, and regulating immune responses. Additionally, the exosomes derived from immune cells, especially the macrophages, play a pivotal role in angiogenesis and osteogenesis. Moreover, macrophages can promote angiogenesis and osteogenesis by shaping the immune microenvironment with anti-inflammatory exosomes derived from M2 macrophages. Since immune cell exosomes are involved in the four stages of osseointegration, functionalized implants can be designed to target immune cell exosomes to regulate the process of osseointegration, which might be an effective way to avoid the aseptic loosening and improve the success of implantation.
